# Severe Acute Respiratory Distress Syndrome (ARDS) or Severely Increased Chest Wall Elastance?

**DOI:** 10.7759/cureus.22541

**Published:** 2022-02-23

**Authors:** Simon Lindner, Daniel Dürschmied, Ibrahim Akin, Simone Britsch

**Affiliations:** 1 Department of Cardiology, Angiology, Haemostaseology and Medical Intensive Care, University Medical Center Mannheim, Mannheim, DEU; 2 European Center for Angioscience (ECAS) and German Center for Cardiovascular Research (DZHK), Partner Site Heidelberg/Mannheim, Mannheim, DEU

**Keywords:** transpulmonary pressure, covid-19, esophageal manometry, extracorporeal membrane oxygenation, acute respiratory distress syndrome

## Abstract

Esophageal manometry can be used to calculate transpulmonary pressures and optimize ventilator settings accordingly. We present the case of a 31-year-old male patient with ataxia-telangiectasia (Louis-Bar syndrome) and a BMI of 20 kg/m^2^, admitted to our intensive care unit for coronavirus disease 2019 (COVID-19) pneumonia. The patient soon required mechanical ventilation; however, there was very poor respiratory system compliance. Cholecystitis complicated the clinical course, and veno-venous extracorporeal membrane oxygenation (ECMO) was initiated as gas exchange deteriorated. Esophageal manometry was introduced and revealed severely increased intrathoracic pressure and chest wall elastance.

## Introduction

All parts of the respiratory system must be considered when mechanical ventilation is initiated in patients with acute respiratory distress syndrome (ARDS). The overall mechanical characteristics are influenced by both the lung and the chest wall [1]. Increased stiffness of the chest wall that surrounds the lung requires higher pressures to ventilate the lung even when the lung itself shows normal mechanical behavior. The pressure that is required for inflation to 1 liter above resting position is defined as elastance. To assess the individual contribution of lung and chest wall to the overall respiratory system elastance, intrathoracic pressures can be obtained by esophageal manometry. The calculated transpulmonary pressures reveal lung and chest wall compliance and can then be used to individualize ventilator settings [2]. Especially in morbidly obese patients, where pleural pressures are elevated due to more weight on the thorax and abdomen, this method has been useful to guide ventilation [3]. However, high chest wall elastance due to stiffness of the thoracic cage or increased intraabdominal pressures can similarly lead to increased pleural pressures [4], without patients showing obvious clinical characteristics.

## Case presentation

A 31-year-old male patient was admitted to our hospital for coronavirus disease 2019 (COVID-19) pneumonia to receive oxygen support. After three days, he was transferred to our intensive care unit due to ARDS of increasing severity. The patient was suffering from ataxia-telangiectasia (Louis-Bar syndrome), a rare neurodegenerative, autosomal recessive disease, which, in this patient, had resulted in paresis of the torso and arms, as well as plegia of the lower extremities. He had, however, been mobile in an electric wheelchair. He received no medication prior to hospital admission and had a BMI of 20 kg/m^2^. Louis-Bar syndrome has a poor prognosis with a median life expectancy of 19-25 years, although there are cases reaching an age of 40 years [5]. In a case series comprising critically ill patients, three of seven patients survived till intensive care unit discharge. One of seven patients had survived for three years post discharge [6]. However, this patient had exceeded the median life expectancy. Thus, a less severe disease progression was assumed. As no other negative prognostic factors were present, neither mechanical ventilation nor extracorporeal membrane oxygenation (ECMO) was contraindicated.

Non-invasive respiratory support was started for our patient with alternation between high-flow nasal cannula and non-invasive full-face mask ventilation. However, tolerance of non-invasive mask ventilation was poor and could not be improved sufficiently by the administration of sedatives. Thus, mechanical ventilation was initiated. Initial empiric positive end-expiratory pressure (PEEP) was set to 10 cmH_2_O, following the ARDS network table [7]. A driving pressure (ΔP_aw_) of 25 cmH_2_O was required to achieve sufficient ventilation, resulting in a tidal volume of 230 ml (4.8 ml/kg predicted body weight (PBW)) and a plateau pressure (P_Plat_) of 35 cmH_2_O. To maintain safer P_Plat_ limits, the PEEP was reduced to 7 cmH_2_O. Prone positioning was performed two hours after intubation. The patient then developed acute abdomen due to acute cholecystitis. To safeguard gas exchange during surgery, veno-venous extracorporeal membrane oxygenation (ECMO) was initiated. With a blood flow of 2.5 l/min and a sweep gas flow of 3 l/min, sufficient gas exchange was achieved. PEEP was set to 10 cmH_2_O in an attempt to prevent de-recruitment, and ΔP_aw_ was reduced to 20 cmH_2_O to achieve a P_Plat_ of 30 cmH_2_O. However, these settings resulted in tidal volumes < 100 ml (< 2.1 ml/kg PBW).

Esophageal pressure (P_es_) monitoring was started (Table 1). End-expiratory P_es_ was 22 cmH_2_O, and end-inspiratory P_es_ was 26 cmH_2_O. The PEEP was adjusted to 22 cmH_2_O to achieve a transpulmonary end-expiratory pressure (PEEP minus end-expiratory P_es_) of 0 cmH_2_O. In contrast, with a PEEP of 10 cmH_2_O, the transpulmonary end-expiratory pressure had been -12 cmH_2_O, probably resulting in end-expiratory alveolar collapse. Inspiratory pressure was then raised to increase tidal volume to approximately 6 ml/kg PBW. A tidal volume of 300 ml (6.3 ml/kg PBW) was accomplished with a P_Plat_ of 41 cmH_2_O and a resulting transpulmonary driving pressure (P_Plat_ minus end-inspiratory P_es_) of 15 cmH_2_O.

**Table 1:** First esophageal pressure measurement, calculated transpulmonary pressures, and ventilator adjustments All pressures reported in cmH_2_O; PBW = predicted body weight

	Ventilator	Esophageal	Transpulmonary	Adjusted ventilator	Transpulmonary
End-inspiratory pressure	30	26	4	41	15
End-expiratory pressure	10	22	-12	22	0
Driving pressure	20		4	19	15
Tidal volume	2 ml/kg PBW			6 ml/kg PBW	

The measurements were repeated five hours later (Table 2). End-expiratory P_es_ was now 19 cmH_2_O, resulting in a transpulmonary end-expiratory pressure of 3 cmH_2_O. End-inspiratory P_es_ was 25 cmH_2_O. The PEEP was reduced to 19 cmH_2_O to restore a transpulmonary end-expiratory pressure of 0 cmH_2_O. To maintain an identical tidal volume, ΔP_aw_ could also be reduced, resulting in a transpulmonary driving pressure of 9 cmH_2_O.

**Table 2:** Second esophageal pressure measurement (after five hours), calculated transpulmonary pressures, and ventilator adjustments All pressures reported in cmH_2_O; PBW = predicted body weight

	Ventilator	Esophageal	Transpulmonary	Adjusted ventilator	Transpulmonary
End-inspiratory pressure	41	25	16	34	9
End-expiratory pressure	22	19	3	19	0
Driving pressure	19		13	15	9
Tidal volume	7 ml/kg PBW			6 ml/kg PBW	

Gas exchange improved consistently over the following days. After open cholecystectomy, a complicated postoperative course developed, with acute renal failure and the need for continuous renal replacement therapy. These complications resulted in a delay in ECMO discontinuation. The ECMO was eventually discontinued after 13 days.

## Discussion

The depicted case demonstrates challenges that can arise from special anatomic features in rare diseases, which can be difficult to assess at the bedside. Esophageal manometry can be used to assess chest wall elastance [4]. Measurements in this case clearly indicate high chest wall elastance, probably due to higher chest wall stiffness and increased intraabdominal pressure due to cholecystitis. Adjustment of respirator settings according to calculated transpulmonary pressures resulted in improved gas exchange. Our case highlights the potential of transpulmonary pressure assessment to maintain lung-protective ventilator settings during ECMO therapy. In ARDS, higher airway pressures may be required when high chest wall stiffness, obesity, or increased abdominal pressures are present [1]. When conventional airway pressure safety limits are used, initial PEEP and ΔP_aw_ can be inadequately low. The resulting de-recruitment poses a direct threat of further lung injury, and hypoventilation can delay the discontinuation of ECMO. Esophageal manometry and transpulmonary pressure calculations can identify optimal airway pressures and thus justify transgression of conventional limits while maintaining, or even improving, lung-protective ventilation.

## Conclusions

Transpulmonary pressure calculations were useful to maintain and even improve lung-protective ventilation under ECMO therapy in this case. Not only morbidly obese patients can show severely elevated esophageal pressures. This patient with a normal BMI showed high esophageal pressure due to a severely increased chest wall elastance. Adjustment of ventilator settings to account for chest wall elastance rapidly improved gas exchange and led to the discontinuation of ECMO.
